# 

**Published:** 1917-02

**Authors:** 


					﻿Vol. XXXII
FEBRUARY, 1917
No. 8
TEXAS
MedieaAUoyrnal
				

